# Effect of motorcycle helmet types on head injuries: evidence from eight level-I trauma centres in Taiwan

**DOI:** 10.1186/s12889-020-8191-1

**Published:** 2020-01-17

**Authors:** Carlos Lam, Bayu Satria Wiratama, Wen-Han Chang, Ping-Ling Chen, Wen-Ta Chiu, Wafaa Saleh, Chih-Wei Pai

**Affiliations:** 1 Emergency Department, Department of Emergency and Critical Care Medicine, Wan Fang Hospital, Taipei Medical University, 111 Xinglong Road, Section 3, Taipei, 11696 Taiwan; 2 Department of Emergency Medicine, School of Medicine, College of Medicine, Taipei Medical University, 250 Wuxing Street, Taipei, 11031 Taiwan; 3 Graduate Institute of Injury Prevention and Control, College of Public Health, Taipei Medical University, 250 Wuxing Street, Taipei, 11031 Taiwan; 4 Department of Epidemiology, Biostatistics and Population Health, Faculty of Medicine, Public Health and Nursing, Universitas Gadjah Mada, JL. Farmako, sekip utara sleman district, Yogyakarta, 55281 Indonesia; 5 Department of Medicine, Mackay Medical College, 46 Zhongzheng Road, Section 3, New Taipei, 25245 Taiwan; 6 Department of Emergency Medicine, Mackay Memorial Hospital, 92 Zhongshan North Road, Section 2, Taipei, 10449 Taiwan; 7 Mackay Medicine, Nursing and Management College, 92 Shengjing Road, Taipei, 11260 Taiwan; 8 Institute of Mechatronic Engineering, National Taipei University of Technology, 1 Zhongxiao East Road, Section 3, Taipei, 10608 Taiwan; 9 Transport Research Institute, Edinburgh Napier University, Edinburgh, Scotland

**Keywords:** Motorcycle helmet type, Head injury, Motorcyclist injury severity

## Abstract

**Background:**

Motorcycle full-coverage helmet use may reduce fatalities and head injuries.

**Methods:**

This retrospective cohort study extracted injury data from eight level-I trauma centres in Taiwan and performed a questionnaire survey to investigate injuries sustained by motorcyclists for the period between January 2015 and June 2017.

**Results:**

As many as 725 patients participated in the questionnaire survey and reported their helmet types or phone use during crashes. The results of multivariate logistic models demonstrated that nonstandard helmet (half or open-face helmet) use was associated with an increased risk of head injuries and more severe injuries (injury severity score ≥ 8). Drunk riding and phone use appeared to be two important risk factors for head injuries and increased injury severity. Anaemia was also found to be a determinant of head injuries.”

**Conclusions:**

Compared to full-coverage helmets, nonstandard provide less protection against head injuries and increased injury severity among motorcyclists.

## Background

Taiwan has an estimated population of 23,571,000 people and an area of 36,197 km^2^. Here, 13,690,684 motorcycles were registered in 2017, resulting in a density of 378 motorcycles/km^2^ [1]. The official statistics of Taiwan [2] demonstrated that in 2016, 51% of road traffic fatalities involved motorcyclists. The head was the most commonly injured body region among motorcyclists in Taiwan [3, 4].

Studies [1, 5–9] have consistently concluded that helmet use is beneficial in preventing head injuries and subsequently reducing injury severity among motorcyclists. By using police-reported crash data in Taiwan, Keng [10] found that helmet use was associated with a decreased odds of deaths among motorcyclists. By linking the National Traffic Crash Data and the National Health Insurance Research Database, Pai et al. [1, 3] reported that in Taiwan, helmeted motorcyclists had a decreased odds of hospitalisation due to head injuries. Furthermore, Chen and Pai [11] indicated that helmet use was beneficial in reducing head or neck injuries among motorcyclists involved in approach-turn crashes. The beneficial effects of helmet use on reducing head or neck injuries have also been well documented in other international studies [6, 7, 12–14]. Nonetheless, several studies have argued that helmet use was associated with an increased risk of neck injuries. For example, Goldstein [15] found that helmet use reduced the risk of head injuries but increased that of neck injuries in the event when speed reached more than 13 mph. Ooi et al. [16] concluded that helmet use reduced the risk of cervical spine injuries in a frontal collision, although there was an increased risk of cervical spine injuries in rear-end, side impact, and skid crashes.

Studies have attempted to examine the effects of different helmet types on head and neck injuries. For example, studies [17–22] have reported that riders wearing open-face or half helmets had an increased risk of sustaining facial and neck injuries compared with riders wearing full-face helmets. Rice et al. [23] concluded that novelty helmet or nonstandard helmet use was associated with an increased risk of fatal injuries compared with full-face helmet use after adjustment for speed and other risk factors. By using emergency room admission data in Taiwan, Yu et al. [24] determined that half-coverage helmets provided poor protection from head injuries. By using Taiwan’s National Head Trauma Registry data, Lam et al. [25] reported that full-coverage helmets provided the best protection from neck injuries.

The findings in most studies indicate that full-coverage helmets are beneficial in reducing both head and neck injuries, whereas several studies have argued that helmet use was associated with an increased risk of neck injuries. To the best of our knowledge, few studies have investigated the effect of the helmet type on motorcyclist injuries in Taiwan, where motorcycle is a primary transportation mode. Some studies [4, 5, 8, 17, 24, 25] were conducted using regional hospital data, which may not provide a thorough insight into the underlying relationship between helmet types and motorcyclist injuries. In Taiwan, where motorcycles are the most common means of transportation, the number of hospitalised motorcycle-related head injuries decreased by 33% following implementation of a universal helmet law in 1997 [26]. This study investigated the effect of full-coverage helmet use on head injuries in Taiwan using data from eight level-1 trauma centers.

## Methods

### Study design and participants

We conducted a retrospective cohort study in which we collected data from two sources: hospitals and individual patients. The 2-year data of inpatients (i.e. between January 2015 and June 2017) before the commencement of our research (June 2017) were retrieved from eight participating hospitals. The following eight hospitals represented at least one of the five administration districts in Taiwan: Cheng Ching Hospital Chung Kang Branch, Kuang Tien General Hospital, Mackay Memorial Hospital Taipei Branch, Mackay Memorial Hospital Tamsui Branch, National Cheng Kung University Hospital, Taipei Medical University-Shuang Ho Hospital, Taipei Medical University-Wan Fang Hospital, and Taitung Mackay Memorial Hospital. All eight hospitals are advanced level-I emergency responsibility hospitals.

To extract the data of motorcyclist patients, causes of injury for patients (in accordance with ICD-9-CM (The International Classification of Diseases, Ninth Revision, Clinical Modification) E-codes: E810.2–3, E811.2–3, E812.2–3, E813.2–3, E814.2–3, E815.2–3, E816.2–3, E817.2–3, E818.2–3, and E819.2–3 and ICD-10-CM codes V21–V29) were used. ICD-9-CM N codes ranging from 800 to 999 that report injury diagnoses were used for extracting injury data. Disease diagnoses according to ICD-9-CM N codes or ICD-10-CM codes were used for extracting data on comorbidities. Information regarding the injury severity score (ISS) was also obtained.

The ICD-9-CM and ICD-10-CM is a modified version of World Health Organization (WHO) ICD-9 and ICD-10 created by The U.S. Department of Health and Human Services to classify diseases and cause of diseases using specific codes. This study used ICD-9-CM codes from 800 to 999 and ICD-10-CM S00 – S99 codes to identify injury diagnoses in the study database. We also used ICD-9-CM E810 – E819 and ICD-10-CM V20 – V29 to identify cause of injuries.

We extracted patients’ ISSs [27] from the participating hospitals: ISS is calculated using the sum of squared of the highest Abbreviated Injury Score (AIS) [28] from three different injured body regions. The AIS classified anatomic body region into six parts: head, face, chest, abdomen, extremities, and external. The AIS assigns score 1 to 6 with higher score indicting a more severe injury.

After extracting patient data from the eight participating hospitals, patients were contacted through telephone, and their consent to participate in our study was obtained. A questionnaire survey was administered to obtain additional data such as the helmet type worn, and riding behaviours. Both consent forms and questionnaires were then posted to patients who agreed to participate in the study. Patients received a convenience store voucher that was worth New Taiwan Dollar (NTD) 300 (approximately US $10) as a compensation for filling and returning the questionnaire to the research team. The following patients were not considered: those who sustained fatal injuries, those who were aged < 18 years, those whose nationality was not Taiwanese, and those who could not read the questionnaire. By law, those aged < 18 years cannot legally ride motorcycles, and therefore were removed from our study. Foreign patients were thought to be unable to read our questionnaires in Chinese, and therefore were not included.

### Variables considered

The following variables were retrieved directly from the participating hospitals: patient sex, age (four groups: < 18, 18–40, 41–64, and ≥ 65 years), drunk riding (no: blood alcohol consumption [BAC] level: ≤ 0.03% or yes: BAC level > 0.03%), time of crash (daytime or evening/night), previous medical history (auditory disease: deafness, Meniere’s disease, or tinnitus; visual illness; myopia, presbyopia, cataract, or xerophthalmia; anaemia; or hypertension), primary injured body regions (head, chest, abdomen, or extremities), and ISS. Four age intervals (< 18, 18–40, 41–64, and ≥ 65 years) were initially considered but combined to two intervals (≥ 65 years v.s. otherwise) to increase its statistical significance in later analyses. Casualties’ previous medical history (including auditory disease: deafness, Meniere’s disease, or tinnitus; visual illness; myopia, presbyopia, cataract, or xerophthalmia; anaemia; or hypertension) was identified by the diagnostic codes from the participating hospitals. For example, the following codes were used for extracting anaemia: ICD-9-CM: 280; ICD-10-CM: D50. These pre-crash diseases were hypothesised to affect motorcyclist injury severity.

Other crucial variables not readily available from the participating hospitals were obtained from questionnaires: helmet style, crash location, protective garments used, crash type, medication before crash, and phone use. Specifications for the following variables were obtained from questionnaires: helmet use (standard: full-coverage helmet or nonstandard: open-face or half-coverage helmet), crash location (downtown or provincial highway), other protective garments (yes: protective boots or jacket were used or no: none), crash type (multivehicle or single-motorcycle crash), medication before crash (yes: had been taking medication before crash or no: none), and phone use (yes: using a phone or no: not using a phone).

### Analysis

The current research focuses on head injuries sustained by motorcyclists. The distribution of head injuries and other injured body regions by a set of variables is firstly reported. Chi-squared test was performed for examining the association between independent variables and injured body regions. To minimise type II errors (failing to reject a false null hypothesis) in variable selection and biased inferences, researchers [29, 30] have suggested that the significant levels can be set much higher than conventional levels (i.e., to values of 0.20 or more). We adopted this cut-off *p*-value of 0.2 for including variables in the multivariate logistic regression model. The conventional cutoff of 0.2 has been commonly employed in the literature (e.g. [11, 31]).

ISS, another injury indicator of our interest, was classified into two levels (ISS < 8 and ISS ≥ 8). Studies (e.g., Atkinson et al. [32]) have considered ISS ≥ 8 as an indicator of severe injuries. Similarly, the distribution of two ISS levels (ISS < 8 and ISS ≥ 8) by a set of variables is firstly reported. Choosing a specific threshold for determining severe injuries is in general arbitrary. Several studies have adopted a wide range of ISS levels for determining severe injuries, including ISS greater than 8 [33], 10 [34], or 16 [35]. We followed the ISS threshold suggested by Palmer [36] who reported that ISS levels between > 7 and > 9 can be considered for determining patients who require hospitalisation or intensive care in ICU.

The Chi-square test was used to examine the significant difference between the independent variables and the dependent variables. We then incorporated any variable with *p* < 0.2 in the chi-square test into the multivariate analysis.

## Results

### Respondent profile

**Fig. 1:**
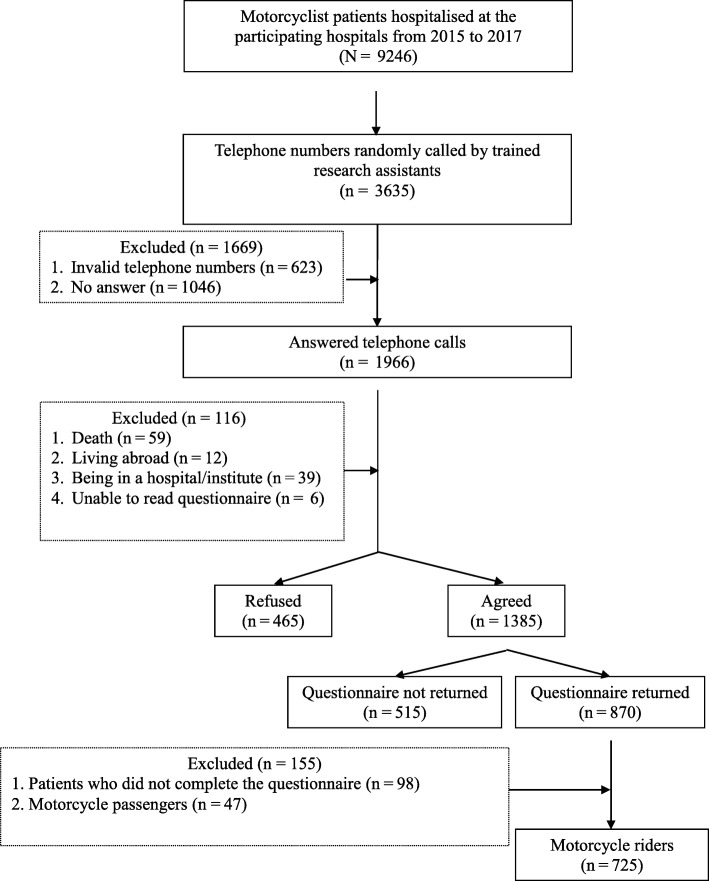
Sample selection process

Figure 1 illustrates the sample selection process from the participating hospitals and the questionnaire survey. As shown in Fig. 1, a total of 9246 motorcyclist patients were enrolled from the eight participating hospitals. Because of time and manpower restriction, we could randomly contact 3635 of the 9246 patients. Among these 3635 patients, 623 had invalid phone numbers and 1046 did not answer calls at all. After excluding these patients (*n* = 1669), 1966 patients remained. Of these 1966 patients or their other family members successfully contacted, 6 could not read the questionnaire, 12 lived abroad, 39 were hospitalised, and 59 passed away. After excluding patients, 1385 patients agreed to participate in our questionnaire survey. Questionnaires were then posted to these 1385 patients, and as many as 870 questionnaires were posted back to our research team. We removed the following cases: patients who did not complete the questionnaire (*n* = 98), and patients who reported to be motorcycle passengers rather than riders (*n* = 47). This yielded a valid sample of 725 patients.

### Helmet style and head or neck injuries

Table 1 lists the distribution of head injuries sustained by motorcyclists according to a set of variables. A vast majority of motorcyclists wore non-standard helmets (82.92%). Approximately 9.80% of all motorcyclist casualties were elderly; 59.16% were males; and 60.38% had full-time jobs. Regarding previous medical history, 3.74, 51.25, 8.34, and 13.41% of all motorcyclist casualties were diagnosed to have auditory diseases, visual illness, anaemic, and hypertension. The majority of motorcycle crashes took place during daytime (75.96%), in downtown areas (94.1%), when riders were wearing protective garments (84.16%), when riders were involved in multiple-vehicle crashes (71.99%). Approximately 4.1% of all motorcyclists were taking medications before crashes; 2.34% were riding under the influence of alcohol; and 3.52% were using mobile phones.

**Table 1:** Distribution of injuries according to a set of independent variables

Variable	Total	Injury location		*P*-value
		Head/neck n(%)	Other body part n(%)	
Helmet type				
1. Non-standard helmet	704 (82.92%)	142 (20.17%)	562 (79.83%)	0.030
2. Standard helmet	145 (17.08%)	18 (12.41%)	127 (87.59%)	
Age				
1. > = 65	84 (9.80%)	21 (25.00%)	63 (75.00%)	0.133
2. otherwise	773 (90.20%)	141 (18.24%)	632 (81.76%)	
Gender				
1. Male	507 (59.16%)	91 (17.95%)	416 (82.05%)	0.390
2. Female	350 (40.84%)	71 (20.29%)	279 (79.71%)	
Occupation				
1. Have full time job	483 (60.38%)	84 (17.39%)	399 (82.61%)	0.186
2. Don’t have full time job	317 (39.62%)	67 (21.14%)	250 (78.86%)	
Previous medical history				
Auditory disease				
1. Yes	31 (3.74%)	7 (22.58%)	24 (77.42%)	0.640
2. No	797 (96.26%)	153 (19.20%)	644 (80.80%)	
Visual illness				
1. Yes	432 (51.25%)	84 (19.44%)	348 (80.56%)	0.724
2. No	411 (48.75%)	76 (18.49%)	335 (81.51%)	
Anaemia				
1. Yes	69 (8.34%)	21 (30.43%)	48 (69.57%)	0.012
2. No	758 (91.66%)	137 (18.07%)	621 (81.93%)	
Hypertension				
1. Yes	111 (13.41%)	27 (24.32%)	84 (75.68%)	0.131
2. No	717 (86.59%)	131 (18.27%)	586 (81.73%)	
Time of traffic				
1. Dawn/night	188 (24.04%)	46 (24.47%)	142 (75.53%)	0.014
2. Daylight	594 (75.96%)	98 (16.50%)	496 (83.50%)	
Traffic location				
1. Downtown area	654 (94.10%)	120 (18.35%)	534 (81.65%)	0.320
2. Provincial highway	41 (5.90%)	5 (12.20%)	36 (87.80%)	
Other protective garments				
1. No	134 (15.84%)	24 (17.91%)	110 (82.09%)	0.747
2. Yes	712 (84.16%)	136 (19.10%)	576 (80.90%)	
Crash type				
1. Multiple vehicle	609 (71.99%)	114 (18.72%)	495 (81.28%)	0.737
2. Single vehicle	237 (28.01%)	42 (17.72%)	195 (82.28%)	
Object type				
1. Fixed object	30 (3.52%)	9 (30.00%)	21 (70.00%)	0.113
2. Non – fixed object	823 (96.48%)	152 (18.47%)	671 (81.53%)	
Medication before riding				
1. Yes	35 (4.10%)	9 (25.71%)	26 (74.29%)	0.301
2. No	818 (95.90%)	153 (18.70%)	665 (81.30%)	
Drunk riding				
1. Yes (BAC level > 0.03%)	20 (2.34%)	9 (45.00%)	11 (55.00%)	0.006
2. No (BAC level < = 0.03%)	833 (97.66%)	153 (18.37%)	680 (81.63%)	
Mobile phone use				
1. Yes	30 (3.52%)	10 (33.33%)	20 (66.67%)	0.037
2. No	823 (96.48%)	150 (18.23%)	673 (81.77%)	

Notably, the percentage of head injuries was higher among motorcyclists wearing non-standard helmets (142; 20.17%) than among those wearing standard helmets (18; 12.41%). Those who had anaemia had a higher risk of sustaining head injuries (30.43%). The percentage of head injuries was higher among motorcyclists involved in dawn or night crashes (24.47%) than among those involved in daytime crashes (16.50%). In total, 45% of those who reported to be drinking alcohol before riding sustained head injuries, which was higher than those indicated otherwise (18.37%). Phone use appeared to be a contributory factor to head injuries: 33.3% of those who reported to be using their phones sustained head injuries.

By using the chi-square test, we determined that the following variables were significantly associated with head injury: helmet type, motorcyclist’s age, occupation, anaemia, hypertension, time of crash, alcohol consumption before riding, and mobile phone use. These variables were then incorporated into stepwise logistic regression models.

Table 2 presents estimation results obtained from stepwise logistic regression models. The estimated parameter for non-standard helmets was significant, suggesting that motorcyclists wearing non-standard helmets were 1.324 times more likely (adjusted odds ratio [AOR] = 1.324; confidence interval [CI] = 1.073–1.634) to sustain head injuries compared with those wearing standard helmets. Other risk factors for head injuries include having anaemia (AOR = 1.985; CI = 1.174–3.357), crashes occurring at dawn or night hours (AOR = 1.7; CI = 1.090–2.651), drinking alcohol before riding (AOR = 1.823; CI = 1.102–3.016), and using mobile phones and riding at the same time (AOR = 2.199; CI = 1.236–3.913).

**Table 2:** Odds of head or neck injuries sustained by motorcyclists

Variable	β	Standard Error	Odds Ratio	95% CI	*P* value
Helmet type					
1. Non-standard helmet	0.2814	0.1264	1.324	1.073–1.634	0.0262
2. Standard helmet	1				
Anaemia					
1. Yes	0.6858	0.2681	1.985	1.174–3.357	0.0107
2. No	1				
Time of traffic					
1. Dawn/night	0.5304	0.2268	1.700	1.090–2.651	0.0194
2. Daylight	1				
Drunk riding					
1. Yes (BAC level > 0.03%)	0.6005	0.2568	1.823	1.102–3.016	0.0196
2. No (BAC level ≤ 0.03%)	1				
Using mobile phone					
1. Yes	0.7880	0.2940	2.199	1.236–3.913	0.0075
2. No	1				

*AIC* 637.394

−2 log likelihood = 625.394

### Helmet style and ISS

Table 3 lists the distribution of severe injuries according to a set of variables. Notably, the percentage of severe injuries (ISS ≥ 8) was higher among motorcyclists wearing non-standard helmets (306; 49.84%) than among those wearing standard helmets (49; 40.16%). As many as 65.79% of elderly riders (aged 65 years or above) exhibited more severe injuries, which was statistically higher than those among their younger counterparts. Those who had anaemia had a higher risk of sustaining more severe injuries (59.32%). The percentage of more severe injuries was higher among motorcyclists involved in multivehicle crashes (50.75%) than among those involved in single-vehicle crashes (40.10%). As many as 68.18% of those who reported to be drinking alcohol before riding sustained more severe injuries, which was higher than those indicated otherwise (47.49%). Phone use appeared to be a contributory factor to more severe injuries: 65.85% of those who reported to be using their phones sustained more severe injuries.

**Table 3:** Distribution of severe injuries (ISS ≥ 8) according to a set of independent variables

Variable	Injury Severity Score		*P* value
	ISS ≥ 8 n (%)	ISS < 8 n (%)	
Helmet type			
1. Nonstandard helmet	306 (49.84%)	308 (50.16%)	0.051
2. Standard helmet	49 (40.16%)	73 (59.84%)	
Age			
1. ≥ 65 years	50 (65.79%)	26 (34.21%)	0.001
2. Other age group	309 (46.26%)	359 (53.74%)	
Sex			
1. Male	221 (49.66%)	224 (50.34%)	0.348
2. Female	138 (46.15%)	161 (53.85%)	
Occupation			
1. Full-time job	196 (46.67%)	224 (53.33%)	0.518
2. No full-time job	136 (49.45%)	139 (50.55%)	
Auditory disease			
1. Yes	12 (52.17%)	11 (47.83%)	0.152
2. No	333 (48.05%)	360 (51.95%)	
Visual illness			
1. Yes	189 (50.13%)	188 (49.87%)	0.839
2. No	165 (46.74%)	188 (53.26%)	
Anaemia			
1. Yes	35 (59.32%)	24 (40.68%)	0.076
2. No	311 (47.26%)	347 (52.74%)	
Hypertension			
1. Yes	46 (47.92%)	50 (52.08%)	0.954
2. No	300 (48.23%)	322 (51.77%)	
Time of traffic			
1. Dawn/night	83 (53.21%)	73 (46.79%)	0.281
2. Daylight	254 (48.29%)	272 (51.71%)	
Traffic location			
1. Downtown area	269 (47.28%)	300 (52.72%)	0.522
2. Provincial highway	20 (52.63%)	18 (47.37%)	
Other protective gears			
1. No	57 (49.57%)	58 (50.43%)	0.767
2. Yes	297 (48.06%)	321 (51.94%)	
Crash type			
1. Multiple vehicle	272 (50.75%)	264 (49.25%)	0.011
2. Single vehicle	79 (40.10%)	118 (59.90%)	
Medication before riding			
1. Yes	19 (63.33%)	11 (36.67%)	0.091
2. No	338 (47.61%)	372 (52.39%)	
Drunk riding			
1. Yes (BAC level > 0.03%)	15 (68.18%)	7 (31.82%)	0.056
2. No (BAC level ≤ 0.03%)	340 (47.49%)	376 (52.51%)	
Using a mobile phone			
1. Yes	27 (65.85%)	14 (34.15%)	0.020
2. No	330 (47.21%)	369 (52.79%)	

By using the chi-squared test, we found that the following variables were significantly associated with severe injury: helmet type, motorcyclist’s age, auditory disease, anaemia, crash type, medication before riding, alcohol consumption before riding, physical and mental status, and using mobile phone use. These variables were then incorporated into stepwise logistic regression models.

Table 4 presents the estimation results of the multivariate logistic regression model relating to severe injuries. The estimated parameter for non-standard helmets was significant, suggesting that motorcyclists wearing nonstandard helmet use were 1.3 times more likely (AOR = 1.300; CI = 1.036–1.631) to sustain more severe injuries compared with those wearing standard helmets. Other risk factors for more severe injuries included elderly motorcyclists (AOR = 2.116; CI = 1.213–3.690), those who got involved in multivehicle crashes (AOR = 1.549; CI = 1.093–2.196), alcohol drinking before riding (AOR = 1.434; CI = 1.094–1.879), and using a mobile phone and riding at the same time (AOR = 2.649; CI = 1.405–5.167).

**Table 4:** Odds of severe injuries (ISS ≥ 8) sustained by motorcyclists

Variable	β	Standard Error	Odds Ratio	95% CI	*P* value
Helmet type					
1. Nonstandard helmet	0.2625	0.1157	1.300	1.036–1.631	0.0241
2. Standard helmet	1				
Age					
1. ≥ 65	0.7496	0.2838	2.116	1.213–3.690	0.0083
2. Other age group	1				
Crash type					
1. Multiple vehicle	0.4379	0.1778	1.549	1.093–2.196	0.0138
2. Single vehicle	1				
Drunk riding					
1. Yes (BAC level > 0.03%)	0.3606	0.1378	1.434	1.094–1.879	0.0091
2. No (BAC level ≤ 0.03%)	1				
Using a mobile phone					
3. Yes	0.9910	0.3322	2.694	1.405–5.167	0.0064
4. No	1				

*AIC* 952.138

−2 log likelihood = 940.138

## Discussion

Studies conducted in Western countries and Taiwan [17, 20–25] have reported that full-coverage helmets protect from head injuries. In line with previous results, our results indicated that standard helmets have a highly protective role. The coverage of entire head and the presence of a chin bar explain why standard helmets provide better protection than nonstandard helmets against head injuries [23]. Our data also revealed that nonstandard helmet use was associated with an increased risk of severe injuries (ISS ≥ 8). The protective effect of full-coverage helmets on head injuries reduced the overall severity of anatomic injuries. In our study, as many as 704 (82.92%) motorcyclist patients wore nonstandard helmets. Our finding here underscores the importance of wearing standard helmets for reducing head injuries, particularly in Taiwan where nonstandard helmets are commonly used (e.g. 704 patients [82.92% of our sample] wore nonstandard helmets).

Our result that nonstandard helmet use was associated with head injuries can be reasonable. This is primarily because first, nonstandard helmets provide lower protection to the heads and chins compared with standard helmets; and if the chin strap is not fully fastened, nonstandard helmets are more likely to be knocked off from a rider’s head compared with standard helmets [37]. Yu et al. [24] reported that motorcyclists wearing loosely fastened helmets had a higher risk of head and brain injuries than did those wearing firmly fastened helmets. In the current research, data on whether helmets were firmly worn were not collected. Further research may attempt to investigate the relationship among injuries, helmet style, and improper helmet use.

Other findings deserve additional discussions here. For example, previous studies have examined the prevalence of mobile phone use in Mexico [38] and Vietnam [39]. Our study contributes to the motorcycle safety literature by concluding that phone use was a determinant of increased injury severity and injuries to the head. Enforcement on prohibiting motorcyclists from using their phones should be tightened.

Regarding drunk riding, studies [1, 11] have reported that riding under the influence of alcohol was associated with hospitalisation due to head injuries and deaths among motorcyclists. Furthermore, intoxicated riders were less likely to wear a helmet and more likely to be speeding and have single-motorcycle crashes compared with nondrinking riders [40]. In accordance with the findings of these studies, we found that drunk riding was a risk factor for increased injury severity and injuries to the head. A better understanding of the relationship among motorcyclist injuries, BAC level, and traffic violation (such as speeding and helmet use) is a fruitful area for further research. The implication of our result here is that instead of motorcycle riders, driving under the influence (DUI) enforcement such as sobriety checkpoints primarily targets car drivers. To reduce alcohol-impaired riding, DUI enforcement should also target motorcycle riders.

Anaemia was found to be an important risk factor for head injuries among motorcyclist patients. Patients with anaemia are generally old and have certain chronic diseases, such as sarcopenia, and one symptom common to most types of anaemia is dizziness [41, 42]. It is likely that a driver fitness to ride a motorcycle is impaired by sarcopenia and dizziness, compelling motorcyclists to have a higher risk of head injuries once an accident has occurred. In Taiwan, by law, those who attempt to take driving- or riding-license tests are mandated to have a health checkup. In addition, labour health checkup is mandatory to employees annually or once per three to five years, depending on employee’s age. The haemoglobin level is routinely checked in these health checkups. Regular screening of the haemoglobin level among those having anaemia can constitute a potential countermeasure.

A primary strength of the current research is the data we obtained from the eight level-I trauma centres, which can be more representative to the whole motorcyclist population than studies that have relied on data from a single hospital or emergency department. However, our study is limited by the binary indicator for the helmet style (standard versus nonstandard). Currently, in Taiwan, there exists no official datasets that contain data for such detailed helmet types among motorcyclist casualties. Further research may attempt to collect more detailed data on helmet types such as tropical helmets (half helmet) or open-faced helmets. Our study is also limited by the fact that all data on patients were obtained from level-I trauma centres, and as a result, a referral bias is inherently present. An important limitation of our study is that fatalities that occurred to patients within hospitals or to those who died before being transported to hospitals were not included in the analysis. Therefore, the effectiveness of helmet types in preventing fatalities and head injuries could not be established. Lastly, there are some missing data in our study which may introduce bias into the results. However, there is no significant difference between participants with and without missing data (*p*-value > 0.05). Therefore, we assumed that the missing data were at least missing at random (MAR) and analysed the remaining data [43].

## Conclusions

Nonstandard helmet use was associated with increased injury severity among motorcyclists after controlling for other risk factors. Furthermore, we concluded that phone use and drunk riding were two risk factors for increased injury severity and head injuries among motorcyclists. We recommend that motorcyclists should be encouraged to wear standard helmets instead of nonstandard helmets. Furthermore, we also recommend an increased enforcement on the use of mobile phone and DUI enforcement should target motorcycle riders and not only car drivers. The need for public awareness on the use of standard helmet, and the role of drunk riding and phone use while riding should be emphasised.

## Data Availability

Because of the contract with Institute of Transportation, Ministry of Transportation and Communications, the original data cannot be made publicly available. As the ethical and legal restrictions from institutional review boards (IRB), data are available for researchers who meet the criteria for access. However, interested researchers may make additional data access requests to the IRB of participating hospitals at: tmujirb@gmail.com for Taipei Medical University; mmhirb82@gmail.com for Mackay Memorial Hospital; irb@ktgh.com.tw for Kuang Tien General Hospital; IRB@ccgh.com.tw for Cheng Ching Hospital Chung Kang Branch; and em73635@mail.hosp.ncku.edu.tw for National Cheng Kung University Hospital.

## Abbreviations

AOR: Adjusted odds ratio
BAC: Blood alcohol consumption
CI: Confidence interval
DUI: Driving under influence
ICD: International classification of disease
ISS: Injury severity score
OR: Odds ratio
US: United States
